# Clinical hematopoietic stem cell-based gene therapy

**DOI:** 10.1016/j.ymthe.2025.04.029

**Published:** 2025-04-24

**Authors:** Tami John, Agnieszka Czechowicz

**Affiliations:** 1 Department of Pediatrics, Division of Hematology, Oncology, Stem Cell Transplantation and Regenerative Medicine, and Center for Definitive and Curative Medicine, Stanford University School of Medicine, Stanford, CA 94305, USA

**Keywords:** hematopoietic stem cell, HSC, gene therapies, clinical trials, gammaretroviruses, lentiviruses, genome editing, conditioning

## Abstract

Hematopoietic stem cell (HSC)-based gene therapies have seen extraordinary progress since their initial conception, now fundamentally transforming the treatment paradigms for various inherited hematologic, immunologic, and metabolic conditions—with additional use cases under exploration. Decades worth of work with advances in viral vector technologies and cell manufacturing have paved the way for HSC gene therapy with marked improvement in the safety and efficiency of gene delivery into HSCs. These have been augmented by the recent rise of innovative genome-editing techniques, particularly using clustered regularly interspaced short palindromic repeats CRISPR-associated proteins (CRISPR-Cas)-based technologies, which have enabled more precise and reproducible genome alterations in HSCs and fostered opportunities for targeted gene modification or gene correction. These breakthroughs have led to the development of many active clinical trials and culminated in the recent federal regulatory-agency approvals of multiple clinical HSC gene therapies for various indications that are now becoming available across different geographies. These treatments aim to offer significant, long-lasting benefits to patients worldwide without the toxicities of alternative treatment approaches. This review explores the history and advancements in HSC gene therapies and provides a comprehensive overview of the latest clinical innovations and cell-therapy products. Further, it concludes with a discussion of the persistent challenges that have limited adoption and potential future opportunities that aspire to enable curative treatment of many different patients through such personalized medicines.

## Graphical abstract

**Figure:**
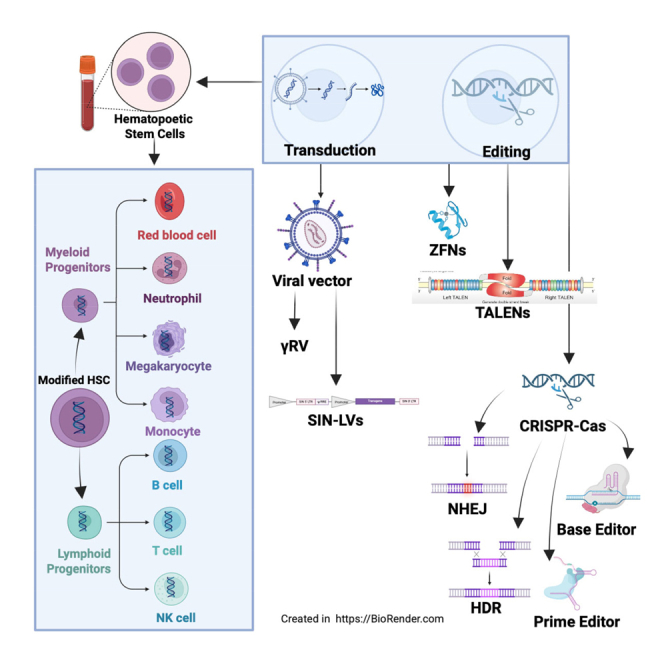

## Introduction

Developing improved treatments for patients has been a long-standing goal for many researchers and physicians. While significant research has been dedicated to understanding clinical ailments, in parallel pioneering scientific approaches have been pursued with an aim for translation into innovative clinical therapies for patients. This is particularly true for many genetic diseases that are caused by inherited DNA mutations with the potential to be effectively treated by overcoming these genetic defects. Genetic modification of hematopoietic stem cells (HSCs) has emerged as a ground-breaking area of research and innovation that began with the goal of treatment of genetic blood and immune diseases and has since exponentially expanded over recent years.

HSCs are unique cells of the bone marrow that are distinguished by their exceptional capacity for long-term self-renewal and their ability to differentiate into all cell types of the blood and immune system. They have been defined by these functional capacities in *in vitro* and *in vivo* assays, which subsequently led to the identification of these cells through unique cell-surface protein combinations and more recently through expression of various unique transcription factors.^1^^,^^2^^,^^3^ HSCs play an important role in the development of hematopoiesis, maintenance of hematopoiesis, and in the response to hematopoietic stress.^4^ These characteristics underscore their immense clinical potential as they can persist and generate a complete hematolymphoid system throughout the lifetime of an individual. Their use has been increasingly leveraged over the past several decades in hematopoietic stem cell transplantation (HSCT), which has become a cornerstone therapy for addressing many different disorders.^5^

While the field of hematopoietic stem cell transplantation (HSCT) originated using normal HSCs from healthy donors, there has been long-standing interest in the use of autologous HSCs as an alternative to address the on-going major challenges of allogeneic HSCT, including reliance on suitable donors and the risks to morbidity and mortality associated with immune suppression and immunologic mismatches. Autologous HSCT eliminates the need for utilizing a donor and the complications related to donor-recipient incompatibility, including infections from immune suppression and graft-versus-host disease.^6^ However, to offer the same possibility of therapeutic benefit for many diseases, the patient’s HSCs must first be modified for enhancement or correction. Autologous HSCT combined with gene modification of HSCs has been developed as a parallel treatment to allogeneic HSCT for many inherited disorders, including severe immunodeficiencies, hemoglobinopathies, and metabolic diseases.^7^^,^^8^^,^^9^^,^^10^ Moreover, this approach has the potential to enable the treatment of disorders where normal HSCs typically cannot provide therapeutic benefit, such as through the utilization of transgenes non-native to HSCs.^11^

**Figure 1:**
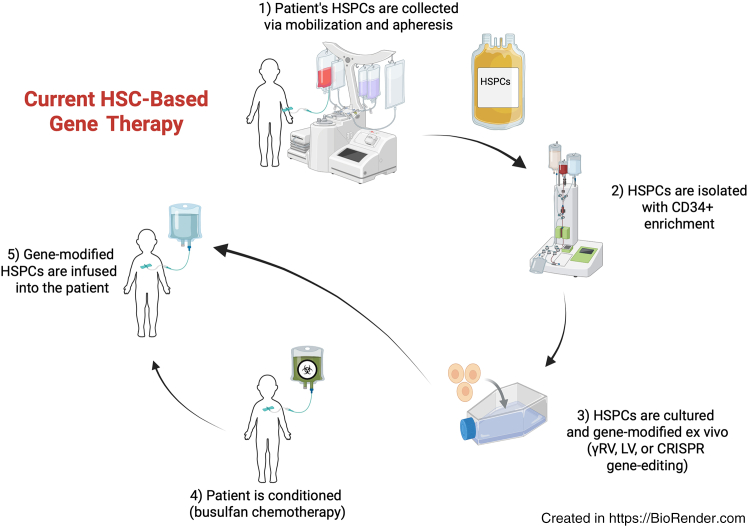
Schematic of current HSC gene-therapy process (1) Hematopoietic stem and progenitors cells (HSPCs) are mobilized and collected via apheresis. (2) HSPCs are then isolated with CD34+ enrichment. (3) The CD34+ enriched cells are then cultured and gene modified *ex vivo*. (4) After manufacturing, the gene-modified HSCs are infused into the patient after conditioning preparation, which most often contains busulfan chemotherapy.

Notably, with autologous gene-modified HSCT, the patients serve as their own donors and their own HSCs are collected, genetically modified *ex vivo*, and reinfused after preparative conditioning chemotherapy (Figure 1).^12^ *Ex vivo* genetic modification of HSCs can be achieved using viral vectors to deliver intended therapeutic genes or through genome-editing techniques that enable precise, site-specific alterations tailored to specific mutations or diseases. While these HSCT approaches provide many potential benefits to patients, they do require manufacturing of personalized therapies, which poses its own unique challenges.

Given the explosion of clinical HSC gene therapies, this review was undertaken to comprehensively assess the state of the field and expose the wide range of clinical trials, drug product approvals, and diverse sponsorships. It examines the current landscape of HSC gene therapy, exploring recent advancements and on-going clinical trials. It begins with an overview of evolving gene transfer and genome-editing methodologies, including both historical approaches and more recent advancements, emphasizing the approaches that have led to the first federal regulatory-agency approvals for HSC-based gene therapies. Next, a comprehensive review was undertaken to compile products with regulatory approvals, appraise current clinicaltrials.gov trials listings, and evaluate other gene-therapy reviews to produce a comprehensive collection of successful clinical studies and active clinical trials with gene-modified HSCs. Finally, the discussion shifts to on-going challenges and concludes with an outlook on the future potential directions for development of these promising therapeutic technologies.

## Mechanisms of HSC gene therapy

To date, gene-modification techniques in HSCs have predominantly focused on autologous *ex vivo* approaches that have followed a common treatment paradigm (Figure 1). This method involves harvesting HSCs from the patient, which can be done through bone marrow harvest or more commonly through apheresis after mobilization of HSCs from the bone marrow into the peripheral blood using drugs such as granulocyte colony-stimulating factor (G-CSF) and/or the CXCR4 antagonistic plerixafor.^12^ After collection, the graft is most often enriched for CD34+ hematopoietic stem and progenitor cells (HSPCs) and then cultured *ex vivo* with genetic modification using vector transduction or genome-editing technologies to overcome or correct the underlying defect. In most cases, the patient then undergoes chemotherapy conditioning to prepare the bone marrow microenvironment by clearance of competing HSPCs, and the genetically engineered cells are then reintroduced into the patient via intravenous infusion whereby they home to the bone marrow to engraft, self-renew, and differentiate into various blood cell types.^13^ This creates a long-lasting reservoir of gene-modified HSCs capable of producing all blood cell lineages, which, over time, alleviates the disorder.

The *ex vivo* gene-modification approach offers a controlled setting for cell manipulation, allowing for thorough assessment of cell function and product characterization prior to transplantation. In most cases the product is cryopreserved after the manufacturing and subsequently thawed immediately prior to use if it meets appropriate product characteristics. This process has now been standardized by many groups with various pre-specified product release criteria that dictate utilization.^14^ This strategy holds the potential to ensure a one-time curative treatment of a patient due to the durable engraftment of standardized, genetically altered HSCs while recognizing that each product is also unique and personalized to the individual from which it was created. Gene therapy using HSCs has already shown great promise for addressing various immune-system disorders, hemoglobinopathies, and metabolic diseases, as shown by the federal regulatory approval for clinical use of several advanced therapy medicinal products (ATMPs).^15^ Notable examples of approved clinical products include Strimvelis for adenosine deaminase deficiency-associated severe combined immunodeficiency (ADA-SCID), Zynteglo and Casgevy for transfusion-dependent β-thalassemia (TDT), Libmeldy for pediatric metachromatic leukodystrophy (MLD), Skysona for early cerebral adrenoleukodystrophy (CALD), and Lyfgenia and Casgevy for sickle cell disease (SCD) (Table 1). These products are now starting to be disseminated through clinical practices, although they have various constraints that have limited their uptake and still require treatment at specialized HSCT centers.

**Table 1:** Approved clinical HSC-based gene-therapy products with federal regulatory-agency approvals

Disease indication	Product: generic (marketed)	Manufacturer	Mechanism of action	Approval agency (year)
ADA-SCID	GSK2696273 (STRIMVELIS)	GlaxoSmithKline/Orchard Therapeutics/Telethon Foundation	ADA gene addition via γRV	EMA (2016)
TDT	betibeglogene autotemcel (ZYNTEGLO)	bluebird bio	βA-T87Q-globin gene addition via lentivirus (LV)	EMA (2019)^a^, USFDA (2022)
MLD	atidarsagene autotemcel (LIBMELDY)	Orchard Therapeutics	ARSA gene addition via LV	EMA (2020), USFDA (2024)
CALD	elivaldogene autotemcel (SKYSONA)	bluebird bio	ABCD1 gene addition via LV	EMA (2021)^a^, USFDA (2022)
SCD	lovotibeglogene autotemcel (LYFGENIA)	bluebird bio	βA-T87Q-globin gene addition via LV	USFDA (2023)
SCD/TDT	exagamglogene autotemcel (CASGEVY)	Vertex Pharmaceuticals/CRISPR Therapeutics	CRISPR-Cas9 knockout of BCL11A enhancer via electroporation	EMA, UK MHRA, NHRAB (2023), USFDA (2023/2024), CDA, SFDA, Swissmedic (2024)

CDA, Canada’s Drug Agency; EMA, European Medicines Agency; NHRAB, National Health Regulatory Agency of the Kingdome of Bahrain; SFDA, Saudi Federal Drug Agency; UK MHRA, UK Medicines and Healthcare Products Regulatory Agencies; USFDA, US Food and Drug Administration.

a Withdrawn at the request of the marketing-authorization holder.

### Original gammaretroviral approaches

While gene-modified HSC products have become more common in recent years, the HSC gene-therapy field has a long-standing history and was initially established in the early 1990s.^16^^,^^17^ Viral vectors for *ex vivo* genetic modification were the gateway into HSC gene therapy and this approach has remained a cornerstone in the field. Viral vectors harness the natural efficiency of viruses in delivering genetic material into cells and hence were used as the initial delivery modalities for gene therapy. However, these vectors, which are derived from wild-type viruses, had to be engineered to remove most viral protein-coding genes, rendering them replication-incompetent and non-infectious to prevent subsequent spread.^18^ As HSCs and their progeny proliferate and divide, DNA-integrating viruses have been commonly utilized in HSC gene therapy.

Over the years, various viral vector systems that are based on native viruses have been utilized in gene-therapy development and clinical trials. These include viral vectors based on the *Retroviridae* family (gammaretroviruses and lentiviruses), adenoviruses, and adeno-associated viruses—with recent efforts also focused on particles derived from these viruses.^19^ Countless groups have been involved in the development of these approaches, and each of these platforms has demonstrated unique benefits and challenges during both preclinical and clinical studies, which has driven their utilization and subsequent further optimization. Among these, gammaretroviral vectors (γRVs) launched the field forward and were the first to be widely tested for clinical gene therapy due to their low immunogenicity, ability to integrate their genome into host cells, and high transduction efficiency in actively dividing cells.^20^ The first regulatory approved gene-modified HSC therapy, Strimvelis for ADA-SCID, utilized a modified γRV (Table 1).^21^^,^^22^ Unfortunately, toxicity concerns have limited and, in most cases, halted γRV use in the clinic. Specifically, γRVs have been found to preferentially integrate near transcriptional start sites and within CpG islands, with a tendency to target proto-oncogenes.^23^^,^^24^ This integration pattern has posed a risk of insertional oncogenesis and adverse events, as was observed in the early X-SCID trials where leukemia developed from the gene-modified HSCs ultimately terminated these efforts.^25^ These safety concerns have driven the development of alternative approaches and vector systems.

### Follow-on lentiviral approaches

The use of lentiviral vectors (LVs) has become the more recent standard approach for HSC-based gene therapy that is now used by many different academic and commercial groups throughout the world. LVs used in clinical applications are typically derived from human immunodeficiency virus (HIV)-1 that is stripped of its structural genes and frequently pseudotyped with a heterologous envelope protein.^19^ These modifications grant LVs the unique ability to transduce quiescent cells, such as HSCs, as they transport viral single-stranded RNA into the nucleus through nuclear pores, resulting in faster transduction and supporting a large packaging capacity enabling delivery of larger genes that are stably integrated into the genome.^19^ Self-inactivating LVs (SIN-LVs), with their improved safety profile and reduced genotoxicity risks, have since become the preferred delivery method in HSC gene therapy—although these are still closely monitored for potentially troubling integrations and dominance of specific gene-modified clones.^26^

While integration sites are still semi-random and generally unpredictable when using LVs, integrations tend to cluster in actively transcribed regions, and both preclinical and clinical studies suggest that LV-integration patterns differ significantly from γRVs without the associated genotoxic risks.^27^ Specifically, LV integrations span megabase-wide genomic areas without accumulating in sharp, isolated peaks targeting single genes—patterns often linked to oncogenesis in other retroviral vectors. Alternative vector systems, such as adenoviruses and adeno-associated viruses, are non-integrating, thereby limiting their use for gene delivery and resulting in limited success in direct HSC applications, although efforts with these viruses are on-going especially with other directed payloads.^19^ Hence, LVs have emerged as the most reliable platform that eases gene transfer into HSCs.

Notably, LV-mediated HSC gene engineering has proved to be a critical tool in clinical practice, enabling curative therapies for a wide range of conditions. Several clinical trials have already demonstrated marked effectiveness with long-term follow-up, culminating in regulatory approvals of advanced therapies for diseases such as TDT,^28^^,^^29^ MLD,^30^ CALD,^31^ and SCD^32^ (Table 1). Additionally, dozens of other lentiviral clinical trials are underway all around the world to develop subsequent therapies for these diseases and others such as for the treatment of Wiskott-Aldrich syndrome (WAS),^33^ leukocyte adhesion deficiency type 1 (LAD1),^34^ Fanconi anemia-A,^35^ and pyruvate kinase deficiency (PKD)^36^ with many follow-on indications. LV technologies have enabled an exponential expansion of clinical HSC gene therapies, which is appreciable through the wide range of gene-therapy trials and sponsors in the space (Table 2).

**Table 2:** On-going clinical trials testing new HSC-based gene-therapy products

Clinical trial registry number	Disease	Intervention	Sponsor	Phase	Study start	Status
**LV-based gene transduction of HSCs for inherited disorders**						

NCT01410825	WAS	autologous CD34^+^ cells transduced with the w1.6_hWASP_WPRE (VSVg) LV	David Williams, Boston Children’s Hospital	I/II	7/1/11	active, not recruiting
NCT01639690	βTDT	autologous CD34^+^ cells transduced with the TNS9.3.55 LV encoding the β-globin gene	San Rocco Therapeutics	I	7/1/12	active, not recruiting
NCT01306019	X-linked severe combined immune deficiency (SCID-X1)	autologous CD34^+^ cells transduced with VSV-G pseudotyped LV CL20- 4i-EF1alpha-hgammac-OPT	NIAID	I/II	9/25/12	recruiting
NCT02757911/NCT01855685	X-linked chronic granulomatous disease (X-CGD)	autologous CD34^+^ cells transduced with the G1XCGD LV	Genethon	I/II	6/24/13	active, not recruiting
NCT02247843	SCD	autologous CD34^+^ cells transduced with the Lenti/G-βAS3-FB LV	Donald B. Kohn, University of California, Los Angeles	I/II	12/1/14	active, not recruiting
NCT02559830	MLD, ALD	autologous CD34^+^ cells transduced with LV encoding the human ARSA (for MLD)/ABCD1 (for ALD) cDNA	Shenzhen Second People’s Hospital	I/II	1/1/15	recruiting
NCT03282656/NCT05353647	SCD	autologous CD34^+^ cells transduced with LV containing a short hairpin RNA targeting BCL11a (GRASP)	David Williams, Boston Children’s Hospital	I/II	2/13/18	active, not recruiting
NCT03311503	SCID-X1	autologous CD34^+^ cells transduced with the rHIV_IL2RGcoG2SCID LV	David Williams, Boston Children’s Hospital	I/II	2/26/18	active, not recruiting
NCT03488394	MPS-IH	autologous CD34^+^ cells transduced with the IDUA LV encoding the human α-L-iduronidase gene	IRCCS San Raffaele	I/II	5/11/18	active, not recruiting
NCT03538899	Artemis-SCID	autologous CD34^+^ cells transduced with the AProArt LV encoding the DCLRE1C gene	University of California, San Francisco	I/II	5/31/18	recruiting
NCT01306019	SCID-X1	autologous CD34^+^ cells transduced with LV encoding the IL2RG gene	Great Ormond Street Hospital for Children NHS Foundation Trust	I	12/21/18	recruiting
NCT03837483	WAS	autologous CD34^+^ cells transduced with the LV encoding WAS cDNA	Fondazione Telethon	III	1/21/19	active, not recruiting
NCT03897361	cystinosis	autologous CD34^+^ cells transduced with pCCL-CTNS or pCDY.EFS.CTNS.T260I LV	University of California, San Diego	I/II	7/8/19	active, not recruiting
NCT03812263	LAD1	autologous CD34^+^ cells transduced with LV encoding the ITGB2 gene	Rocket Pharmaceuticals, Inc.	I/II	8/30/19	active, not recruiting
NCT04797260	RAG1-SCID	autologous CD34^+^ cells transduced with the pCCL.MND.coRAG1.wpre LV	Leiden University Medical Center	I/II	11/12/19	recruiting
NCT03964792	SCD	autologous CD34^+^ cells transduced with the GLOBE1 LV encoding the βAS3 globin gene	Assistance Publique - Hôpitaux de Paris	I/II	11/12/19	active, not recruiting
NCT04069533/NCT04248439	Fanconi Anemia, subtype A	autologous CD34^+^ cells transduced with LV carrying the FANCA gene	Rocket Pharmaceuticals	II	11/28/19	active, not recruiting
NCT04201405	Mucopolysaccharidosis type IIIA, Sanfilippo Syndrome type A	autologous CD34^+^ cells transduced with LV containing the human SGSH gene	University of Manchester	I/II	1/7/20	active, not recruiting
NCT03818763	hemophilia A	autologous CD34^+^ cells transduced with LV encoding the ITGA2B gene promoter for ectopic expression of human B-domain-deleted factor VIII	Medical College of Wisconsin	I	4/29/20	recruiting
NCT04105166	PKD	autologous CD34^+^ cells transduced with LV carrying the codon optimized red cell pyruvate kinase gene	Rocket Pharmaceuticals	I	7/6/20	active, not recruiting
NCT05265767	hemophilia A	autologous CD34^+^ cells transduced with LV encoding a novel coagulation factor VIII transgene	Christian Medical College, Vellore, India	I	4/1/22	active, not recruiting
NCT05432310	ADA-SCID	autologous CD34^+^ cells transduced with the EFS-ADA LV encoding the ADA enzyme	University of California, Los Angeles	I/II	1/4/23	recruiting
NCT05762510/NCT05757245	βTDT	autologous CD34^+^ cells transduced with the GMCN-508B LV (LentiRed)	First Affiliated Hospital of Guangxi Medical University	early I	2/22/23	recruiting
NCT05207657	p47 Autosomal p47 AR-CGD	autologous CD34^+^ cells transduced with the pCHIM-p47 LV encoding the NCF-1 gene	Great Ormond Street Hospital for Children NHS Foundation Trust	I/II	3/20/23	recruiting
NCT05757245	αTDT	autologous CD34^+^ cells transduced with the GMCN-508A LV	First Affiliated Hospital of Guangxi Medical University	I	5/8/23	recruiting
NCT05860595/NCT06219239	βTDT	autologous CD34^+^ cells transduced with the βA-T87Q-globin gene LV (KL003)	Institute of Hematology and Blood Diseases Hospital, China/Kanglin Biotech	N/A	5/23/23	recruiting
NCT05665166	mucopolysaccharidosis type II, Hunter syndrome	autologous CD34^+^ cells transduced with the CD11B LV encoding human IDS tagged with ApoEII	University of Manchester	I/II	6/1/23	recruiting
NCT05071222	Artemis-SCID	autologous CD34^+^ cells transduced with the G2ARTE LV expressing the DCLRE1C cDNA	Assistance Publique - Hôpitaux de Paris/Genethon	I/II	7/19/23	recruiting
NCT05776173/NCT05773729/NCT05015920/NCT06465550	βTDT	autologous CD34^+^ cells transduced with LV encoding the βA-T87Q-globin gene (BD211)	Shanghai BDgene	N/A	8/10/23	recruiting
NCT06149403	MPS-IH	autologous CD34^+^ cells transduced with LV encoding the human IDUA gene (OTL-203)	Orchard Therapeutics	III	12/11/23	recruiting
NCT06308159	βTDT	autologous CD34^+^ cells transduced with LV encoding functional hemoglobin subunit beta (HBB) gene	Lantu Biopharma	I/II	5/11/24	recruiting
NCT05745532/NCT06655662	βTDT	autologous CD34^+^ cells transduced with LentiHBBT87Q LV (HGI-001)	Shenzhen Hemogen	I	6/12/24	recruiting
NCT06253507	p47 AR-CGD	autologous CD34^+^ cells transduced with the pCCLCHIM-p47 LV	NIAID	I/II	6/25/24	enrolling by invitation
NCT06399107	SCD	autologous CD34^+^ cells transduced with the BAH243 LV	Essen Biotech	I/II	8/1/24	recruiting
NCT06364774	βTDT	autologous CD34^+^ cells transduced with the ALS20 LV encoding human βA-T87Q-globin	Children’s Hospital of Philadelphia	I/II	2/1/25	recruiting

**LV-based gene transduction of HSCs for HIV and cancer therapeutics**						

NCT01961063/NCT02337985	AIDS-related non-Hodgkin lymphoma	autologous CD34^+^ cells transduced with rHIV7-shI-TAR-CCR5RZ LV	City of Hope Medical Center	I	11/20/15	active, not recruiting
NCT02797470	HIV-related lymphoma	autologous CD34^+^ cells transduced with LV encoding CCR5 shRNA/TRIM5alpha/TAR decoy	AIDS Malignancy Consortium	I/II	6/23/16	active, not recruiting
NCT04849910	CD33+ acute myeloid leukemia or myelodysplastic syndrome	allogeneic CD34^+^ cells modified using CRISPR-Cas9 editing to remove the CD33 protein to enable increased use of CD33-ADC (Mylotarg)	Vor Biopharma	I/II	12/16/21	recruiting
NCT05052957	glioblastoma multiforme	autologous CD34^+^ cells transduced with LV carrying the P140K MGMT gene to protect hematopoiesis from chemotherapy	Leland Metheny	II	1/20/23	recruiting
NCT05922384	HIV-1 with non-Hodgkin lymphoma	autologous CD34^+^ cells transduced with the lenti-7shRNA vector	Affiliated Hospital of Guangdong Medical University	N/A	7/5/23	recruiting
NCT06716853	metastatic renal cell carcinoma	autologous CD34^+^ cells transduced with LV encoding the interferon-α2 gene	Genenta Science	I/II	10/22/24	recruiting

**CRISPR-based gene editing of HSCs for inherited disorders**						

NCT04443907	SCD	autologous CD34^+^ cells modified using CRISPR-Cas9 editing to induce mutations in HBG1/2 promoter	Novartis Pharmaceuticals	I	8/25/20	active, not recruiting
NCT04853576	SCD	autologous CD34^+^ cells modified using AsCas12a to induce mutations in HBG1/2 gene promoters (EDIT-301)	Editas Medicine	I/II	5/4/21	active, not recruiting
NCT05444894	βTDT	autologous CD34^+^ cells modified using AsCas12a to induce mutations in HBG1/2 gene promoters (EDIT-301)	Editas Medicine	I/II	4/29/22	recruiting
NCT06041620	βTDT	autologous CD34^+^ cells modified with CRISPR-Cas12b editing to induce mutations in HBG1/2 gene promoter	Institute of Hematology and Blood Diseases Hospital, China/Kanglin Biotech	N/A	8/31/23	recruiting
NCT06300723	SCD	autologous CD34^+^ cells modified with CRISPR-Cas9 editing the BCL11A gene (BRL-101)	Bioray Laboratories	N/A	7/29/24	Enrolling by invitation

**CRISPR-based gene correction of HSCs for inherited disorders**						

NCT04819841	SCD	autologous CD34^+^ cells gene modified using CRISPR-Cas9 and HDR-editing to convert HbS to HbA (nula-cel)	Kamau Therapeutics	I/II	11/15/21	recruiting
NCT04774536	SCD	autologous CD34+ cells gene modified by the CRISPR-Cas9 ribonucleoprotein at the sickle allele (CRISPR_SCD001)	Mark Walters, University of California, San Francisco	I/II	9/18/24	recruiting

**Base or prime editing of HSCs for inherited disorders**						

NCT05456880	SCD	autologous CD34^+^ cells modified with base editing to induce mutations in HBG1/2 gene promoters (BEAM-101)	Beam Therapeutics	I/II	8/30/22	recruiting
NCT06107400	hemoglobin H-constant spring disease	autologous CD34^+^ cells modified with base editing to repair the CS mutation of HBA2 gene (RM-004)	The 923rd Hospital of Joint Logistics Support Force of People’s Liberation Army	early I	10/8/23	recruiting
NCT06328764/NCT06291961	βTDT	autologous CD34^+^ cells modified with base editing to induce mutations in HBG gene promoter (CS-101)	CorrectSequence Therapeutics	early I/I	3/19/24	recruiting
NCT06325709	X-CGD	autologous CD34^+^ cells modified with base editing to repair CYBB missense gene mutations	NIAID	I/II	4/17/24	recruiting
NCT06559176	p47 AR-CGD	autologous CD34^+^ cells modified with prime editing to correct the delGT mutation in NCF1(PM359)	Prime Medicine	I/II	11/1/24	recruiting

**Other HSC-based gene-modification approaches**						

NCT02500849	HIV-1	autologous CD34^+^ cells modified using ZFN SB-728mR to disrupt CCR5	City of Hope Medical Center	I	3/10/16	active, not recruiting
NCT06017869	Pearson syndrome	autologous CD34^+^ cells enriched with allogeneic placental-derived mitochondria	Minovia Therapeutics	I	7/31/23	recruiting

Review performed from www.clinicaltrials.gov for recruiting, enrolling by invitation, and active clinical trials (accessed on 31 December 2024). Trials are grouped by treatment indication and then organized by study start date. Long-term follow-up studies are not included. NIAID, National Institute of Allergy and Infectious Diseases; MPS-IH, mucopolysaccharidosis type I, Hurler syndrome; βTDT, transfusion-dependent β-thalassemia; p47 AR-CGD, recessive chronic granulomatous disease.

### Newer genome-editing approaches

The development of programmable gene-modification agents, including nucleases, base editors, and prime editors, has further revolutionized the gene-therapy field, enabling genome editing by precise genetic modifications in DNA, which ultimately enables more controlled expression of therapeutic genes. These approaches have enabled non-viral gene modification of HSCs. While gene editing began with zinc-finger nucleases (ZFNs) and transcription activator-like effectors (TALENs), they have dramatically exponentiated in the era of CRISPR-associated proteins (CRISPR-Cas)-based technologies, which have both eased use and expanded access.^37^ CRISPR-based technologies utilize site-specific endonucleases to create double-stranded breaks at specific DNA sites, which then recruit DNA-repair mechanisms to introduce targeted changes.^38^^,^^39^ Depending on the application, these modifications may lead to gene disruption, correction, or insertion of genetic material. Although more specific than random integration, off-target effects are possible, and efforts are underway to both monitor and minimize these through improved gene-engineering approaches. Many variations of these technologies are now in development, which are rapidly being implemented in many preclinical settings with advancing clinical development.

Notably, the first CRISPR-Cas-based gene-editing therapy designed to treat both SCD and TDT, Casgevy, was approved in 2024 by regulatory agencies for clinical use in multiple countries.^40^^,^^41^^,^^42^^,^^43^ This product utilizes electroporation of HSPCs with CRISPR-Cas9 and guide RNA disrupting the transcriptional repressor gene, *BCL11A*, to ultimately disinhibit production of fetal hemoglobin.^44^^,^^45^ Additional therapies for β-globinopathies are in development that edit the γ-globin genes (*HBG1*/*HBG2*) to mimic hereditary persistence of fetal hemoglobin (Table 2). One such therapy targets *HBG1*/*HBG2* promoters by inducing mutations with CRISPR-AsCas12a.^46^ Another approach uses CRISPR-guided base editing where a coupled deaminase enzyme chemically changes a base letter to disrupt *HBG1*/*HBG2* and disinhibit fetal hemoglobin production.^47^^,^^48^

Other genome-editing technologies target correction of monogenic diseases by applying CRISPR technology and homology-directed repair (HDR) to knockin a donor DNA template.^49^^,^^50^ In the setting of SCD, this technology aims to correct the single-nucleoside polymorphism of the β-globin gene (*HBB*) causing sickling hemoglobin, and various HDR-driven repair techniques remain under investigation^51^^,^^52^ (Table 2). Base editors are also in development to correct monogenic diseases, which may be a more specific correction approach.^53^ One such possibility involves base editing of the sickle point mutation in *HBB* using an adenine base editor correcting it to the non-pathogenic β-globin variant, hemoglobin G-Makassar.^54^ Finally, prime editors have further recently been developed to correct the p47phox variant that causes the most common autosomal recessive form of chronic granulomatous disease (CGD)^55^ (Table 2). As these newer technologies are still in clinical development or have had less clinical follow-up, their long-term effects are less understood. However, many more genome-editing clinical trials are likely to be initiated in the future with both academic and industry sponsorship given the reprogrammable nature of CRISPR-based technologies, which eases development of follow-on products.

## Safety considerations

Prioritizing the safety of HSC gene therapy is critical for ensuring long-term clinical success—and this success is a result of both the cell modification and the conditioning. Safety concerns remain regarding toxicity of conditioning chemotherapy, engraftment of functional and durable gene-modified stem cells, detriment of off-target effects, and gene-therapy-associated mutagenesis. To date, HSC gene therapy has, in almost all use cases, utilized genotoxic conditioning, most notably with the myelotoxic alkylating agent busulfan. Other genotoxic agents have less commonly been utilized, including melphalan and treosulfan.^56^^,^^57^ The goal of preparative chemotherapy is to “empty” the bone marrow, thereby enabling enhanced engraftment of the gene-modified HSC graft. However, this genotoxic conditioning is known to cause severe side effects, including cytopenias, mucositis, sinusoidal obstructive syndrome/veno-occlusive disease (SOS/VOD), infertility, pulmonary fibrosis, and other organ damage.^58^ Notably, this genotoxic conditioning also increases risk of both hematologic and solid-tumor malignancies.

### Engraftment and therapeutic efficacy

Engraftment of gene-modified HSCs is crucial for bone marrow recovery and therapeutic efficacy. Reasons for suboptimal engraftment encompass manufacturing failures such as inefficient gene transfer or gene editing of HSCs and/or generally poor graft function of gene-modified HSCs. Regulatory guidance around manufacturing of quality drug product includes minimum release criteria for potency (specific to each drug product), sterility, purity metrics, and a minimum post-manufacturing cell dose included in post-marketing prescriber information. The US Food and Drug Administration (USFDA) prescribing label for Casgevy and Lyfgenia recommends a minimum of 3 × 10^6^ CD34^+^ cells/kg^40^^,^^59^ and for Zynteglo and Skysona a minimum of 5 × 10^6^ CD34^+^ cells/kg^60^^,^^61^ for infusion.

Viral-vector gene therapies require efficient gene delivery and adequate expansion of the gene-modified HSCs. Lessons learned from the early trials with gene addition of *HbAT87Q* in SCD include the importance of vector transduction efficacy and minimum threshold for healthy modified HSCs to achieve engraftment and therapeutic efficacy. Protocol changes after the first cohort treated with the drug product BB305 for SCD included peripheral blood mobilization and apheresis collection (as opposed to bone marrow harvest) to significantly increase HSC collection volume and manufacturing updates that improved vector transduction efficiency resulting in higher vector copy number (VCN) detected in blood cells, increase in gene product (HbAT87Q%), and reduction of vaso-occlusive events (primary endpoint).^62^

The precision and technical success of HSC gene editing stems from successful on-target editing of repopulating HSCs with avoidance of detrimental off-target editing. The common DNA-repair mechanism non-homologous end joining (NHEJ) results in nucleotide insertions and deletions (INDEL) formation in the target gene rendering it non-functional or knocked out.^63^ Early gene editing using ZFNs (drug product BIVV003) for TDT resulted in early engraftment and efficacy but loss of target effect and clinical efficacy after 1 year, indicating, at least in part, the potential failure to achieve significant on-target editing of long-term repopulating HSCs.^64^^,^^65^ In contrast, current application of HSC gene editing with CRISPR-Cas9 with Casgevy has resulted in efficient on-target editing of *BCL11A* and resultant increase and sustained fetal hemoglobin production up to 2 years has thus far been published.^45^^,^^66^

Newer investigative approaches such as gene correction via utilization of complex coupling of DNA repair with HDR and integration of donor DNA template require further study for on- and off-target editing efficiency. The success of such an approach is dependent on successful on-target activity and avoidance of both off-target activity and significant INDEL formation. In SCD, for example, significant INDEL formation within *HBB* compromises success of the therapy as it is non-corrective but is expected to be secondarily disease forming of β-thalassemia.^50^ The first patient treated with nulabeglogene autogedtemcel (gene correction of *HBB* in SCD) experienced delayed red blood cell (RBC) and platelet engraftment, and therapeutic targets of sickle hemoglobin (HbS) and standard adult hemoglobin (HbA) at 1 year were low at 4.5% and 12.5%, respectively, despite complete clinical resolution of acute sickle-related events. Bone marrow evaluation showed 80% of cells were edited with only 2% corrected and a high frequency of INDELs (75%) indicating poor correction efficacy of engrafted stem cells.^52^ Resolution of sickle-related acute events appears related to significant fetal hemoglobin production, which may be a hematopoietic stress response post HSC-based therapy, acquired thalassemia, or other causes. The associated clinical trial is actively recruiting (Table 2). Newer technologies in clinical development include base editors and prime editors, which are expected to exhibit very efficient on-target editing, although stem cell health and long-term engraftment of gene-modified HSCs will be of interest in clinical trials.^67^

### Mutagenesis

Early progress in the HSC gene-therapy field faced significant setbacks due to safety concerns, particularly the risks of insertional mutagenesis with viral vector systems. This was initially observed in the X-SCID clinical trial where several patients treated with γRVs subsequently developed lymphocytic leukemia.^68^^,^^69^ This was linked to vector integration near the LMO2 gene, resulting in its upregulation and likely initiation of the observed blood cancers. These malignant transformations temporarily halted the trial, although it later resumed for patients lacking other treatment options, showcasing the unmet needs in this area. Similarly, other gene-therapy trials that followed experienced holds, delays, and increased scrutiny due to the potential risk of leukemia, although many eventually moved forward after assessing the risk-benefit balance in favor of patients.^70^ Leukemias were not limited to this X-SCID trial, as malignant transformations emerged in other trials using γRVs for conditions such as ADA-SCID,^71^ X-CGD,^72^ and WAS,^73^ which were also noted to be caused by vector integration near other proto-oncogenes.

Most recently, the LV-based product Skysona has received attention for a growing number of associated myeloid malignancies. Patients treated in the clinical trials that led to regulatory approvals of this therapy are in follow-up with a median of 6 years, and malignancies appear to be due to clonal vector insertions within oncogenes with evidence of clonal evolution.^74^ The LV in this therapy includes a single intact internal promoter-enhancer to drive expression of *ABCD1* in multiple cell types, including microglia and macrophages, which likely contributes to increased risk for tumorigenesis. A high proportion of patients have evidence on integration site analysis (ISA) of persistent oligoclonality with insertion in a known oncogene, and a number of patients have developed myelodysplasia/myeloid leukemia with clonal evolution of somatic genetic defects primarily due to vector insertion within oncogenes (e.g., *SMG6*, *MECOM*, *CCND2-AS1*, *MPL*, and *C6ORF10*).^75^ The contribution of mobilization and chemotherapy regimens to leukemogenesis is unclear, although six of seven reported cases of leukemia occurred in the study group having received primarily GCSF without plerixafor for HSC mobilization and busulfan-fludarabine chemotherapy as opposed to busulfan-cyclophosphamide. Despite the known oncogenesis risk, Skysona remains USFDA approved due to continued perceived benefit over other therapies, and the evaluation of a different LV construct for CALD is underway in China (Table 2). So far as we know, no cases of insertional oncogenesis have been reported in other approved LV-based gene-transduction therapies with differing LV constructs (Libmeldy, Zynteglo, Lyfgenia) or in the enhancer-deleted γRV therapy for ADA-SCID (Strimvelis). It is important to note that not all leukemias in HSC gene therapy arise from vector-induced mutagenesis. For example, leukemic blasts examined in patients treated in early trails resulting in Lyfgenia lacked vector-derived genetic material, suggesting that dysplasia was instead related to conditioning, graft fitness, and/or the pathophysiology of SCD rather than the vector.^76^^,^^77^

In response, the field has focused on advancements in various areas to improve safety, including viral vector engineering, particularly with SIN-LVs that lack strong promoter-enhancer sequences, and development of an enhanced understanding of integration sites through rigorous preclinical safety testing and subsequent clinical monitoring algorithms. Given the aforementioned history of leukemias, the USFDA recommends monitoring at regular intervals and over the long term (15 years) after receipt of therapies involving vector integration.^78^ Specifically, clinical monitoring for dysplasia and clonal hematopoiesis is recommended, including periodic blood counts and bone marrow evaluations with pathologic review, flow cytometry, cytogenetic assessment, and targeted next-generation sequencing (NGS) panel testing searching for evolving gene variants with malignant potential. In addition, ISA using linear amplification-mediated polymerase chain reaction (LAM-PCR) is a surveillance strategy that allows for identification and mapping of points of DNA integration for the purpose of mutagenesis risk prediction and monitoring.^79^^,^^80^ Integration-site analysis testing is not yet commercially available, and provisions for monitoring in the clinical setting have been the responsibility of the sponsor.

Concerns about genotoxicity are not restricted to viral vector gene therapy but extend to the gene-editing field. Emerging evidence with genome-editing approaches points to potential for unexpected oncogenic on-target events, such as large deletions, crossover events, distal lesions, and chromosomal translocations, that can compromise genomic integrity and potentially serve as initial carcinogenic hits in long-lived stem cells.^81^ Additionally, the activation of the p53-mediated DNA damage response by CRISPR-induced double-strand breaks may favor cells with mutations in the p53 pathway, raising oncogenic risks.^82^ Highly sensitive and specific on- and off-target detection systems involving *in silico* computation tools and/or NGS techniques are crucial for ensuring accuracy and safety of gene editing.^83^^,^^84^^,^^85^ The field is rapidly evolving with new technologies, and no broad consensus on the most appropriate assessment pathway currently exist. The USFDA similarly currently recommends monitoring of dysplasia and evolving clonal hematopoiesis for up to 15 years after gene-editing therapies,^78^ which also typically includes periodic blood counts and bone marrow evaluations with pathologic review, flow cytometry, cytogenetic assessment, and targeted NGS panel testing. To date, CRISPR-based gene-editing approaches in clinical application have resulted in on-target precision editing without reports of significant off-target events, and cancers have not been described, although long-term monitoring is required and on-going.

## Diverse potential clinical applications of HSC gene-therapy products

HSC gene therapy originated with the correction of monogenic inherited blood and immune disorders with the restoration of function of the missing gene and subsequent proteins. This began with SCID, and quickly expanded to β-thalassemia and SCD and subsequently metabolic disorders. This continues to be the major application of gene-modified HSC products with all seven approved products focused on treatment of monogenic disorders—and an additional >40 active gene-therapy clinical trials directed at treating this category of disorders (Table 2).

While many of these products are aimed at the same more-common inherited genetic diseases, the diversity of genetic disorders that are being approached through such treatments is rapidly expanding. Given the ability of HSCs to make all types of blood and immune cells with diverse functions and activities, many additional use cases of HSC gene therapy have been considered and are now being developed. Specifically, various groups have been working to modify HSCs to produce proteins that are deficient in inherited disorders that may not be native to HSCs. A notable example of this is for hemophilia A, where factor VIII is naturally produced by sinusoidal endothelial cells in the liver. However, through genetic modification, HSCs have been generated to acquire this new function of producing factor VIII in resulting downstream progeny via myeloid^11^^,^^57^ or platelet-directed delivery of a factor VIII transgene.^86^^,^^87^ Other use cases for HSC gene therapy have developed in the areas of acquired diseases such as HIV and cancer. Specifically, various groups have been exploring gene modification of HSCs to protect against HIV infection and/or to produce anti-viral agents.^88^ Similarly, HSC gene therapy has been explored to protect HSCs from chemotherapy, allow for use of higher-dose treatments for cancer, and/or enable use of other targeted immunotherapies ranging from antibody-drug conjugates to chimeric antigen receptor T cells that target antigens that are shared by cancer cells and healthy HSC-derived cells.^89^^,^^90^^,^^91^ In addition, the diverse roles of HSCs that include generation of microglial and various transporter proteins enable use in the treatment of many disorders ranging from mucopolysaccharidoses to cystinosis.^92^^,^^93^ Finally, there are on-going efforts to modify HSCs through administration of mitochondria, which can augment mitochondrial DNA for the correction of mitochondrial diseases such as Pearson’s syndrome.^94^^,^^95^ As HSC gene therapy continues to become safer and more accessible, additional use cases such as these are expected to develop with time, and additional disease indications are likely to be explored from cancer predispositions to autoimmune diseases, which could utilize autologous, allogeneic, or induced pluripotent stem cell (iPSC)-derived HSCs. New guidance from the USFDA on platform technology designation may further increase the potential to treat many monogenic inherited blood and immune disorders where the prevalence of a single disorder has historically been deemed too low to justify new product development.^14^

## Future advances

While much progress has been made in the HSC gene-therapy field lending competition to allogeneic HSCT, additional improvements are still needed to improve efficacy, increase safety, broaden gene therapy to more diseases, and expand access. On-going improvements include modifications in HSPC mobilization, better selection of the most potent cells, and improved *ex vivo* culture and processing.^96^^,^^97^^,^^98^ Additionally, better viral vectors such as new generations of SIN-LV more precise gene-editing approaches including base and prime editors and improved manufacturing strategies are being explored and utilized.^15^^,^^99^ These could enable improved products for disorders where gene therapy has already been investigated and developed, and this could open new approaches for treatment of many other disorders.

The known risks and toxicities related to myeloablative chemotherapy beget the need for less toxic bone marrow preparative strategies. One promising strategy harnesses the potential for non-genotoxic preparative regimens, the most promising of which utilizes monoclonal antibody (mAb)-based conditioning to selectively deplete the residual bone marrow HSCs. Initial efforts focused on HSC-targeted conditioning have centered on targeting the protein CD117 (c-Kit) due to the exquisite sensitivity of HSCs to stem cell factor (SCF), the HSC growth cytokine that binds to CD117, and subsequent combination approaches and antibody-drug conjugates are in on-going development.^100^^,^^101^^,^^102^ To date, only CD117 mAb conditioning has been advanced to the clinic using both antagonistic antibodies and antibody-drug conjugates with other targets being pursued with radioisotopes. Further, these agents have thus far only been utilized clinically with allogeneic HSCs,^103^^,^^104^ although, in preclinical settings, they have been shown to enable engraftment of gene-modified HSCs.^105^^,^^106^ Additional targets have also been pursued, and various proteins, including CD27, CD45, CD90, CD110, CD184, and CD300f, have demonstrated efficacy as conditioning agents in preclinical models.^101^^,^^107^^,^^108^^,^^109^ These have not yet been advanced to the clinic, as they likely require effector moieties that could have additional toxicity, and, given the distribution of these proteins, there is increased potential concern for depletion of other cells. More recently, several groups have also shown that HSCs can be engineered to have a selective advantage to conditioning mAbs to enable selective engraftment of gene-modified HSCs.^110^^,^^111^ One final strategy to avoid toxic chemotherapy uses HSC-mobilizing agents like GCSF, plerixafor, and/or a VLA-4 inhibitor to move HSCs and free space in the bone marrow niche at the time of infusion of gene-modified stem cells.^112^^,^^113^^,^^114^ To date, these non-chemotherapy techniques have resulted in suboptimal engraftment of HSCs, and combination of practices may be necessary to reduce and eventually eliminate preparative toxic chemotherapy.

Finally, the advent of improved genomic modification has opened doors for the potential of *in vivo* genome modification in HSCs, enabling personalized and potentially curative treatments without *ex vivo* manipulation. A variety of promising delivery tools are being explore for such use. Given their efficacy in other organ systems, nanoparticles (NPs) have emerged as a possible tool for delivering therapeutic agents. Lipid NPs (LNPs) have also shown the potential to reduce electroporation-induced toxicity and improve the efficiency of edited cell yields.^115^ Leveraging learnings from HSC-based mAb conditioning, such NPs could eventually be equipped with targeting motifs specific to HSCs.^116^ Alternatively, modified viral vectors or viral-derived particles could be utilized for delivery to HSCs.^117^^,^^118^ Such approaches could enable targeted *in vivo* gene modification; however, these approaches are challenged by novel potential off-target effects such as modification of non-hematopoietic cell types, which could result in additional risks and toxicities.^119^ Notably, some commercial entities have announced in the media strategic planning for translation of *in vivo* gene editing to the clinical space, although, to date, no clinical trials have been registered in clinicaltrials.gov.

## Real-world challenges and sustainability

Despite the promising clinical outcomes and approval of several HSC-based gene therapies, widespread utilization has not yet occurred. The availability and pricing of these therapies combined with on-going toxicity concerns has been a large limitation to use. However, this reflects the complexity of *ex vivo* HSC gene therapy, which, in its current state, is highly individualized, technically demanding, and has on-going unacceptable risks. These factors limit access to many of these treatments to resource-rich areas in high-income countries and are not without challenges for reimbursement by public health systems and private payors.^120^ Further, the difficulties in maintaining financial solvency early during commercialization have hindered access to these therapies around the world with products such as Strimvelis and others such as those for WAS and CGD deprioritized by their manufacturer.^121^ Strimvelis, for example, was taken off the market due to limited commercial utilization and strategic reprioritization by Orchard Therapeutics, although the Italian research charity Telethon announced in 2023 continued manufacture and distribution of this HSC gene therapy.^122^ Other products such as Zynteglo and Skysona were withdrawn from the European market by their manufacturer bluebird bio after achieving regulatory approval due to reimbursement discrepancies.^123^ Additionally, in 2024, Editas Medicine announced the transition from *ex vivo* HSC gene editing despite the success of their clinical trials to *in vivo* gene editing.^124^ Indeed, many for-profit programs have halted or closed during or after the clinical trial period to prioritize programs that support financial solvency and generate more significant revenue.

While scientific and clinical therapeutic interest remain robust, the high cost of HSC gene-therapy clinical trials and the commercialization process have been prohibitive for translation to post-approval revenue, and the future state of the field is in peril.^125^ Despite their potentially transformative clinical benefit, current healthcare payor systems in the US are not equipped to support a shift in cost from the current model of regular and paced costs over many years to an ultra-high up-front payload.^120^^,^^126^ This is especially true for rare and unique diseases where patient volume does not support rapid or lucrative profit. Similarly, for universal healthcare systems, the one-time ultra-high individual cost is not feasible and appraisal of clinical benefit over less costly allogeneic HSCT remains a challenge. Finally, on-going risks of current therapies has continued to drive additional scientific innovation, which creates competition, limits use of early iterations of effective gene therapies, and makes it difficult to recuperate development costs.

Addressing the financial barriers and supporting scientific ingenuity worldwide could pave the way for more realistic, accessible, and equitable gene-therapy treatments.^127^ This could be accomplished by decreasing costs and complexities associated with current *ex vivo* cell manufacturing, eliminating genotoxic conditioning, which causes a need for extensive hospitalization, re-structuring reimbursement pathways for ultra-high-cost one-time therapies, and supporting scientific innovation within the field utilizing local resources in diverse medical systems, especially where disease propensity is highest.^128^ Further, reducing regulatory hurdles that add expense and challenge efficient translation of new technology worldwide could ease the development of such therapies. Standardizing various aspects of HSC-based gene therapy to decrease development costs and enable platform-based products that could be used across various disease indications may be imperative. Finally, strategic organization of philanthropic, for-profit, and non-profit efforts could enable access and support exploration of non-traditional routes for providing these treatments.^127^^,^^129^

## Conclusions

The field of HSC gene therapy has made significant strides over the past three decades, culminating in the approval of several ATMPs by various regulatory bodies such as the European Medicines Agency (EMA) and USFDA allowing their commercial availability. The first approval of Strimvelis was a major milestone for the field for the treatment of ADA-SCID, utilizing a γRV to deliver the therapeutic gene. Zynteglo, designed for patients with TDT, followed with its LV transduction technology encoding a β-globin transgene, addressing a genetic deficiency in these patients. Finally, in 2024, Casgevy became the first HSC gene therapy using CRISPR gene editing to be approved in multiple countries, including the US, European Union, United Kingdom, Canada, Switzerland, Saudi Arabia, and Bahrain for patients with TDT and SCD. Notably, translation of these therapies to the post-approval space has been difficult, with many drugs being withdrawn from the market due to the significant cost and complexity of current HSC gene therapies. Nonetheless, numerous HSC gene-therapy trials utilizing LVs and CRISPR technology are actively progressing with hopes of approval and commercialization (Table 2).

Looking ahead, integrating innovative technologies and focusing on early development strategies that balance clinical efficacy with economic viability will be essential to expand the availability of these treatments. Bridging the gap between research and clinical application will require a commitment to addressing both scientific and financial challenges. Given the notable scientific advances in the field, the commitment of many investigators and regulatory agencies, and the recognition of the challenges that these many disorders bring to patients, we are optimistic for what the next decade holds for the clinical HSC gene-therapy field.

## Acknowledgments

The authors acknowledge helpful discussions with many colleagues at the Stanford University Center for Definitive and Curative Medicine (CDCM) as well as those from throughout the field.

## Author contributions

A review on this specific topic was solicited by the journal. T.J. wrote and revised the manuscript and designed the figures. A.C. planned and participated in writing and revising the manuscript, created the tables, and contributed to the figures.

## Declaration of interests

T.J. has participated as an advisory board member and received consulting fees from bluebird bio, Vertex Pharmaceuticals, and BiolineRx. She is a medical monitor for BMT CTN 2001 GRASP and BMT CTN CRISPR_SCD001 studies, for which she receives compensation. T.J. is the Stanford site principal investigator of clinical trials for genome editing sponsored by Beam Therapeutics (NCT04443907). T.J. has no direct financial interest in this therapy. A.C. discloses financial interests in the following entities working on gene-therapy- and antibody-based conditioning approaches: Beam Therapeutics, Editas Medicines, GV, Inograft Biotherapeutics, and Prime Medicines. In addition, she is an inventor on antibody-based conditioning patents licensed to Jasper Therapeutics, Gilead Sciences, Inograft Biotherapeutics, and Magenta Therapeutics and has received sponsored research funding from Jasper Therapeutics, Rocket Pharmaceuticals, and STRM.Bio.

## Declaration of generative AI and AI-assisted technologies in the writing process

During the preparation of this work, the authors used Stanford GPT-4o-mini ⁠to create an HSC gene-therapy outline. After using this service/tool, the author(s) built on this and extensively edited, adding in the content that is included in this review. They take full responsibility for the content of the publication.
